# Targeting Regulation of Macrophage to Treat Metabolic Disease: Role of Phytochemicals

**DOI:** 10.1111/cpr.70012

**Published:** 2025-03-05

**Authors:** Zeting Ye, Yanlin Li, Xiaolin Yang, Chenglin Li, Rui Yu, Guangjuan Zheng, Zuqing Su

**Affiliations:** ^1^ State Key Laboratory of Traditional Chinese Medicine Syndrome The Second Affiliated Hospital of Guangzhou University of Chinese Medicine Guangzhou China; ^2^ Guangdong Provincial Key Laboratory of Chinese Medicine for Prevention and Treatment of Refractory Chronic Diseases The Second Clinical College of Guangzhou University of Chinese Medicine Guangzhou China; ^3^ Guangdong‐Hong Kong‐Macau Joint Lab on Chinese Medicine and Immune Disease Research The Second Clinical College of Guangzhou University of Chinese Medicine Guangzhou China; ^4^ State Key Laboratory of Dampness Syndrome of Chinese Medicine The Second Affiliated Hospital of Guangzhou University of Chinese Medicine Guangzhou China

**Keywords:** macrophage, metabolic syndrome, metaflammation, phytotherapy

## Abstract

Metabolic syndrome encompasses a cluster of predictive metabolic risk factors, including obesity, insulin resistance, dyslipidemia, hyperglycemia and hypertension. It is strongly associated with the development of type 2 diabetes and cardiovascular disease. Given the increasing morbidity and mortality associated with metabolic syndrome, along with the limited availability of drug treatments, it is high time to investigate the pathogenesis of this condition and explore potential pharmacotherapies. Macrophages, well‐known innate immune cells, play an essential role in maintaining tissue immune homeostasis and multiple physiological processes, including glucose and lipid metabolism, oxidative stress and inflammation. Emerging evidence indicates that the effects of macrophages in metabolic syndrome are linked to macrophage‐mediated metaflammation. Phytochemicals derived from natural plants have been shown to exert therapeutic effects on metabolic syndrome by modulating macrophage function. In this review, we sort out the role of macrophage‐mediated metaflammation in the pathogenesis of metabolic syndrome and summarise potential phytochemicals that target macrophages for the treatment of this condition.

## Graphical abstract

One‐third of the world's population is affected by metabolic syndrome. Complicated pathogenesis and limited drugs cause the growing prevalence of metabolic syndrome. Macrophage‐mediated metaflammation is closely associated with the development of metabolic syndrome. The role of phytochemicals targeting macrophages in the treatment of metabolic syndrome has received increasing attention.

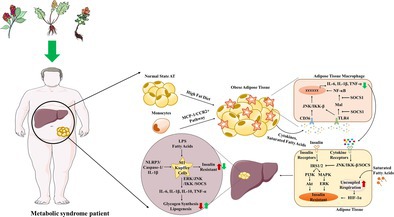

**Abbreviations**
ABCA1: ATP‐binding cassette transport A1
ABCG1: ATP‐binding cassette transport A1 G1
ACAT1: acetyl‐CoA acyltransferase‐1
ACC: Acetyl‐Coenzyme A carboxylase
ANT2: ADP/ATP translocase 2
ATF3: activating transcription factor 3
C1QTNF1: C1q tumour necrosis factor‐regulated protein 1
CETP: cholesterol ester transfer protein
CIC: citrate carrier
CPT: carnitine palmitoyltransferase
CPT1 and CPT2: carnitine palmitoyl transferases 1 and 2
CRR2: chemokine (C‐C motif) receptor 2
FAO: fatty acid oxidation
FoxO1: Forkhead box O1
G6PD: Glucose‐6‐phosphate dehydrogenase
HDL‐C: high‐density lipoprotein cholesterol
HDLs: high‐density lipoproteins
IDH: isocitrate dehydrogenase
IKK‐β: IκB kinase β
IL‐1β: interleukin‐1β
IRS: interleukin‐1 receptor‐associated kinase‐M; IRS; insulin receptor substrates
JNK: c‐jun N‐terminal kinase
LCAT: lecithin cholesterol acyltransferase
LDLs: low‐density lipoproteins
LPL: lipoprotein lipase
LPS: lipopolysaccharide
LXR: liver X receptor
Mal: MyD88‐adaptor‐like
MAPK: mitogen‐activated protein kinase
MCP‐1: monocyte chemoattractant protein 1
Mfn2: mitochondrial mitofusin 2
MS: metabolic syndrome
NADPH: nicotinamide adenine dinucleotide
NCEH1: neutral cholesterol ester hydrolase 1
NF‐κB: nuclear factor κB
Nrf2: nuclear factor erythroid 2‐related factor 2
PHD: prolyl hydroxylase domain
PHD2: prolyl‐hydroxylase 2
PPARs: Peroxisome proliferator‐activated receptors
ROS: reactive oxygen species
RXR: retinoid X receptor
SHD: succinate dehydrogenase
SR‐B1: scavenger receptor B class type 1
TIGAR: TP53‐induced glycolysis and apoptosis regulator
TLR4: Toll‐like receptor 4
VLDLs: very low‐density lipoprotein

## 1 Introduction

Patients with metabolic syndrome (MS) are characterised by obesity, insulin resistance, hyperglycemia and hyperlipidemia, and they are also at high risk of developing type 2 diabetes, cardiovascular disease, and cancer [1, 2]. According to the newest definition from the National Cholesterol Education Program—Adult Treatment Panel III (NCEP‐ATP III), MS is characterised by obesity, which is diagnosed when waist circumference exceeds 102 cm for men or 88 cm for women. This condition is accompanied by two or more of the following disorders: fasting plasma glucose levels greater than 5.6 mmol/L (100 mg/dL) or a diagnosis of type 2 diabetes; high‐density lipoprotein cholesterol (HDL‐C) levels less than 1.03 mmol/L (40 mg/dL) in men or 1.29 mmol/L (50 mg/dL) in women, or receiving low HDL‐C medication; blood triglycerides levels more than 1.7 mmol/L (150 mg/dL) or receiving medication for elevated triglycerides; and blood pressure more than 130/85 mmHg or taking medication for hypertension [3]. With the globalisation of the western lifestyle, the incidence of global MS has increased rapidly in recent years. In China, the prevalence of MS is approximately 33.9%, while in the United States, it was 34.7% [4]. A modelling analysis has estimated that the global prevalence of MS is about 2.8% in children and 4.8% in adolescents [5]. Moreover, although MS is a disease with complex pathogenesis, high‐fat diet‐induced metaflammation plays a key role in the pathogenesis of MS [6, 7]. Compared with paradigmatic acute inflammation, metaflammation is characterised by chronic, persistent, and low‐grade inflammation, and the subsequently released pro‐inflammatory mediators have been implicated as one of the drivers of MS [8].

Macrophages are innate immune cells that play a key role in adjusting the balance between homeostasis and disease, including tissue and organ development, defence against pathogen invasion, inflammatory response and tissue repair [9]. During human growth and development, it is substantiated that macrophages originate from a progenitor in the yolk sac and evolve into macrophages with specialised functions that are distributed in diverse tissues [10, 11]. Furthermore, it is known that M0 macrophages can polarise to M1 or M2 (M2a, M2b, M2c and M2d) in response to different activators [12, 13]. For example, in obese adipose tissue, excess free fatty acids activate macrophage polarisation toward the pro‐inflammatory M1 phenotype, producing large amounts of pro‐inflammatory factors that impede insulin signalling [14]. Growing evidence shows that hyperactivation of M1 macrophages plays a crucial role in the process of metaflammation that promotes the development of obesity [15], type 2 diabetes [16], and MS [17].

The complex pathogenesis of MS and limited pharmacotherapies have led to an increasing prevalence of MS, suggesting that drugs developed on the basis of elucidated pathogenesis have failed to achieve better efficacy, which has prompted us to investigate new pathogenic mechanisms of MS and candidate drugs based on this mechanism. Recent advances in research suggest that high‐fat diet‐induced macrophage‐mediated metaflammation is one of the keys to the development of MS, which is receiving increasing attention [18]. Therefore, it is essential to review the mechanism of macrophage‐mediated metaflammation in MS and summarise the phytomedicines for treating MS by targeting macrophages, providing a reference for studies and treatments that target macrophage polarisation to prevent and cure MS.

## 2 Metabolic Reprogramming of Macrophages

### 2.1 Macrophage Polarisation

Macrophages are innate immune cells derived from haematopoietic stem cells with great plasticity properties that can be polarised from M0 macrophages to different macrophage subtypes, thus exerting a variety of physiological functions, such as phagocytosis of pathogens and metabolites, presentation of antigens, secretion of chemokines, inflammatory or anti‐inflammatory cytokines and growth factors [19, 20]. Furthermore, macrophages are delivered throughout the body and reside in different organs to maintain tissue homeostasis, such as Kupffer cells in the liver, Langerhans cells in the skin and microglia in the brain [19, 21, 22].

Generally, depending on differences in activation conditions, macrophages can be categorised as two types: pro‐inflammatory M1 and anti‐inflammatory M2. Pro‐inflammatory M1 macrophages are activated by lipopolysaccharide (LPS) or cytokines such as IFN‐γ and GM‐CSF, which release pro‐inflammatory cytokines such as interleukin‐1β (IL‐1β), IL‐6, and tumor necrosis factor alpha (TNF‐α), and engage in the pro‐inflammatory response. Anti‐inflammatory M2 macrophages are polarised by cytokines such as IL‐4 and IL‐13 released from TH2 cells, thus secreting anti‐inflammatory cytokines such as IL‐10 and TGF‐β to participate in inflammation suppression and tissue repair [23].

### 2.2 Macrophage Metabolic Reprogramming

**FIGURE 1:**
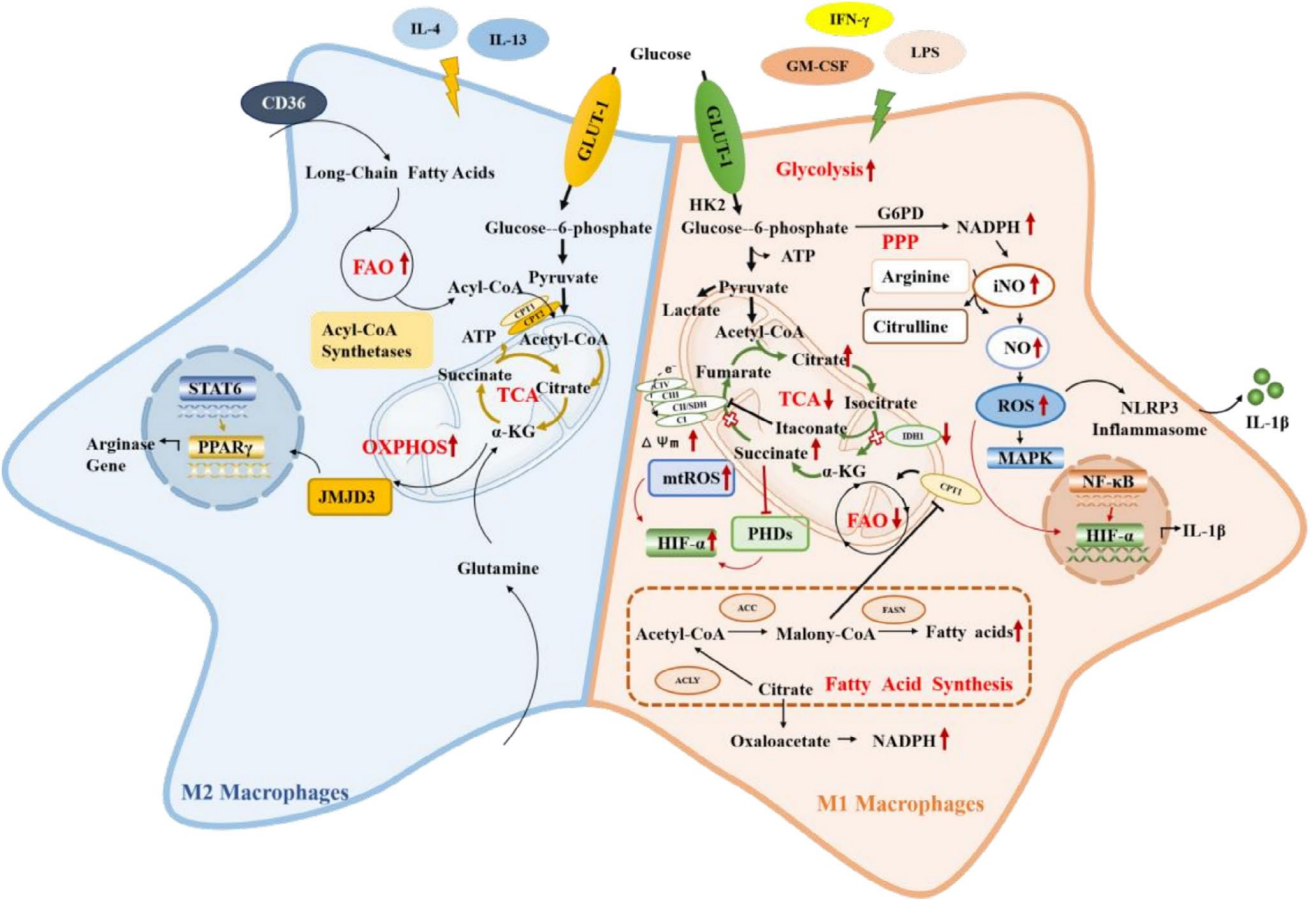
Macrophage metabolic reprogramming.

The phenotypic transition of macrophages depends on changes in the microenvironment, including cytokines and growth factors, and metabolic reprogramming plays a very important role in this process. The metabolism from M0 to M1 presents enhanced aerobic glycolysis, pentose phosphate pathway activity, and fatty acid synthesis, but suppresses mitochondrial oxidative phosphorylation and damages the tricarboxylic acid cycle. In contrast, the metabolism from M0 to M2 presents an intact tricarboxylic acid cycle, stable oxidative metabolism, and increased fatty acid oxidation. This phenomenon of changes in cellular bioenergetics in M1 and M2 is called “metabolic reprogramming” (Figure 1).

#### 2.2.1 Metabolism in M1 Macrophages

In M1 macrophages, metabolism is characterised by increasing the levels of glycolysis and the pentose phosphate pathway, and damaging mitochondrial oxidative phosphorylation [24, 25]. Conventionally, M1 macrophages rely on aerobic glycolysis for ATP generation, even in the presence of enough O_2_, similar to the metabolism of tumour cells, the so‐called Warburg effect theory [26].

During glycolysis in M1 macrophages, extracellular glucose is first delivered across the cell membrane by transport proteins (such as glucose transporters GLUT‐1 and GLUT‐3), and then phosphorylated into glycolytic intermediates (e.g., glucose‐6‐phosphate) by hexokinase [27]. Since glycolysis is an inefficient way to produce ATP, and thus maintaining the balance of cellular energy metabolism requires the delivery of abundant glucose into the intracellular space, the stability of GLUT‐1 is obviously important in M1 macrophages. Furthermore, research on GLUT‐1 inhibitors has also confirmed that the expression of GLUT‐1 is obviously related to the activity of glycolysis in M1 macrophages. For example, BAY‐876, a GLUT‐1 inhibitor, can significantly inhibit glucose intake, glycolytic proton flux rate and lactate production by depressing glycolysis and reducing TNF secretion in CD4^+^ T cells and macrophages, but an increase in the rate of oxidative phosphorylation‐mediated ATP production was also documented [28]. These investigations suggest that GLUT‐1 can be a promising target for the regulation of M1 macrophage‐mediated inflammation. Given the positive role of M1‐type macrophages in anti‐inflammatory, antimicrobial and tumour suppression, it will be worthwhile to investigate how to moderately target GLUT1 to inhibit M1 polarisation [29].

Glucose‐6‐phosphate dehydrogenase (G6PD), a rate‐limiting enzyme of the pentose phosphate pathway that catalyses glucose‐6‐phosphate into 6‐phosphogluconolactone and produces nicotinamide adenine dinucleotide (NADPH), serves as a critical metabolic hub between glycolysis and the pentose phosphate pathway [30]. Growing evidence shows that G6DP bioactivity is highly positively correlated with glycolytic activity in M1 macrophages [25]. Ham and his colleagues have demonstrated that overexpression of G6DP in M1 macrophages can elevate the production of reactive oxygen species (ROS)/RNS by stimulating various pro‐oxidative enzymes, such as iNOS and NADPH, and further activate the p38 mitogen‐activated protein kinase (MAPK) and nuclear factor κB (NF‐κB) signalling pathways to increase the expression of pro‐inflammatory cytokines, including IL‐1β, IL‐6, and TNF‐α [31]. What's more, in the pentose phosphate pathway, NADPH serves as a precursor molecule for iNOS synthesis and further promotes the production of intracellular NO and ROS, resulting in the activation of the NLRP3 inflammasome [32]. Subsequently, the NLRP3 inflammasome can induce pro‐caspase‐1 self‐cleavage and activation, thereby promoting IL‐1β secretion [33]. What's more, iNOS can oxidise arginine to NO and citrulline, which will be further catalysed by a variety of synthases (e.g., argininosuccinate synthase and argininosuccinate lyase) to produce arginine when exogenous arginine is insufficient, thus creating a vicious cycle [34].

Furthermore, in M1 macrophages, mitochondrial oxidative phosphorylation and the tricarboxylic acid cycle are disrupted, which prompts macrophages to switch toward aerobic glycolysis to acquire sufficient energy [32]. In M1 macrophages, the tricarboxylic acid cycle is separated into two parts: (1) The first metabolic break is the accumulation of citrate, which is caused by the down‐regulation of the enzymatic activity of isocitrate dehydrogenase (IDH), which catalyses the oxidative decarboxylation of isocitrate to α‐ketoglutarate, resulting in a significant increase in citrate [34]. Citrate is an essential intermediate of the tricarboxylic acid cycle and an important inhibitor of glycolysis, which is synthesised from oxaloacetate and acetyl‐CoA by citrate synthase in the mitochondrial matrix [35]. Generally, the citrate accumulated in the mitochondria of M1 macrophages is exported into the cytoplasm and accumulates in the cytoplasm via the mitochondrial citrate carrier SLC25A1 [36]. It is worth noting that accumulated citrate in the cytosol can amplify the inflammatory response, while accumulated citrate in mitochondria can perform anti‐inflammatory and antioxidant effects. Cytoplasmic citrate can be catalysed into fatty acids and prostaglandin by cytoplasmic enzymes such as ATP‐Citrate lyase, acetyl‐CoA carboxylase, and fatty acid synthase, which can further participate in macrophage‐mediated inflammatory responses [37, 38]. What's more, cytoplasmic citrate is also converted into oxaloacetate, and thus increases the level of NADPH, which generates ROS through NADPH oxidases [36]. However, mitochondrial citrate can promote decarboxylation of *cis*‐aconitate to produce itaconate, thus activating nuclear factor erythroid 2‐related factor 2 (Nrf2) to inhibit IL‐1β gene transcription, and activating glutathione and activating transcription factor 3 (ATF3) to decrease the level of ROS and negatively regulate pro‐inflammatory cytokines such as IL‐6 [39]. (2) The second metabolic break is caused by decreasing the enzymatic activity of succinate dehydrogenase (SHD), which is a key enzyme that promotes succinate oxidation to fumarate, further providing electrons for the respiratory chain in the inner mitochondrial membrane [40]. The enzymatic activity of SHD can be limited by the elevated itaconate in the first break, resulting in the accumulation of succinate [39]. Furthermore, succinate can stabilise HIF‐1α by inhibiting cytosolic prolyl hydroxylase domain (PHD) enzyme activity and increasing mitochondrial ROS production, which further increases the production of the inflammatory cytokine IL‐1β [41, 42]. Normal mitochondrial membrane potential is a prerequisite for mitochondrial oxidative phosphorylation and ATP production [43, 44, 45]. LPS induces mitochondrial membrane potential hyperpolarisation and further leads to reverse electron transport, resulting in stabilising HIF‐1α through increasing mitochondrial ROS production and accumulating the level of succinate to inhibit PHD enzyme activity [44]. HIF‐α is composed of three subunits: HIF‐1α, HIF‐2α and HIF‐3α, which is also an essential transcription factor for macrophage polarisation [46]. First, HIF‐1α has been confirmed to promote glycolysis and increase inflammatory cytokine release [47]. Second, LPS activates NF‐κB and PI3K/AKT signalling pathways to increase GLUT‐1 expression and stabilise HIF‐1α, and HIF‐1α in turn further boosts GLUT‐1 expression to enhance glycolysis in macrophages [48, 49]. However, some evidence has shown a different response between HIF‐1α and HIF‐2α in macrophage polarisation. For example, HIF‐1α induces iNOS gene expression to increase NO synthesis in M1 macrophages, while HIF‐2α increases the expression of the arginases I gene to suppress NO generation in M2 macrophages [50, 51].

M1 macrophage metabolism is also characterised by enhanced fatty acid synthesis. In the tricarboxylic acid cycle, citrate can be exported to the cytoplasm via the mitochondrial citrate carrier (CIC) and then catalysed by ATP‐Citrate lyase to Acetyl‐Coenzyme A, which serves as a precursor for fatty acid synthesis. With boomed glycolytic flux and increased Krebs cycle break in M1 macrophages, accumulated citrate can activate Acetyl‐Coenzyme A carboxylase (ACC), which converts Acetyle‐Coenzyme A to malonyl‐CoA and disturbs fatty acid oxidation via inhibiting CPT1 [52]. Impaired fatty acid oxidation can increase phosphorylation of IκB, MAPK and c‐Jun, and then exacerbate palmitate‐induced inflammation in M1 macrophages [53]. These studies have suggested that increased fatty acid synthesis and impaired fatty acid oxidation are associated with M1 macrophage activation.

#### 2.2.2 Metabolism in M2 Macrophages

In contrast, M2 macrophages produce abundant ATP mainly through the OXPHOS pathway rather than the aerobic glycolysis pathway. Compared with M1 macrophages, M2 macrophages possess an integrated tricarboxylic acid cycle. Meanwhile, M2 macrophages can metabolise fatty acids and glutamine to provide fuels for the tricarboxylic acid cycle and OXPHOS. Several studies have validated that fatty acid oxidation (FAO) is significantly necessary for M2 macrophage polarisation [54]. The carnitine palmitoyltransferase (CPT) system is an important mediator of FAO, responsible for the transport of long‐chain fatty acids into the mitochondrial matrix, where they are catalysed by acyl‐CoA synthetases to generate acyl‐CoA, which is degraded to acetyl‐CoA via β‐oxidation with the help of carnitine palmitoyltransferases 1 and 2 (CPT1 and CPT2) [55, 56]. Divakaruni and colleagues have demonstrated that etomoxir, a CPT‐1 inhibitor, can not only suppress IL‐4‐induced macrophage polarisation by inhibiting long‐chain fatty acid oxidation, but also hinder M2 macrophage differentiation by depleting intracellular CoA [57]. Peroxisome proliferator‐activated receptors (PPARs) are another key regulatory protein for FAO, including α, β/δ, and γ subtypes. It is well known that the activation of the STAT6/PPARγ/PGC‐1β pathway plays a critical role in M2 macrophage polarisation [58]. In addition, α‐KG via glutaminolysis supports M2 macrophage polarisation by Jmjd3‐dependent metabolic and epigenetic reprogramming and inhibits M1 macrophage‐induced pro‐inflammatory processes by restraining the NF‐κB signalling pathway [59].

## 3 The Role of Macrophages in the Development of MS

Although the pathological process of MS is sophisticated and yet to be fully elucidated, research on the pathogenesis of MS has progressed over the past few decades. Genetic factors, lifestyle, and environmental factors such as excess caloric intake and a lack of physical exercise have been confirmed as important contributors to the development of MS [60], and the potential pathogenesis of MS includes insulin resistance, dysfunction of lipid metabolism and oxidative stress. Recent studies have found that macrophage polarisation is present in all of the above mechanisms, which seems to suggest to us that macrophages are a key factor in triggering the development of MS.

### 3.1 Dysfunction of Lipid Metabolism

Obesity, especially abdominal obesity, is one of the vital diagnostic criteria for MS [61]. In obese individuals, body fat ectopic distribution is the predominant factor that increases the risk of metabolic disease, rather than body total fat mass [62]. Due to a long‐term high‐fat diet, the adipose tissues are unable to deal with excessive fat accumulation due to dysfunction of lipid metabolism and damaged insulin resistance. Excess free fatty acids will be delivered to other tissues, such as the liver, skeletal muscle and heart, known as ectopic fat accumulation [63, 64]. New research suggests that macrophages have the ability to capture and metabolise lipids, which may be one of the key mechanisms by which macrophages are involved in the development of metabolic diseases.

#### 3.1.1 Lipid Metabolism

In normal physiological states, the body's lipids are derived from dietary lipids and endogenous lipids, which are degraded or biosynthesised into metabolites such as triglycerides, fatty acids, and cholesterol, and stored [65, 66]. The dietary lipid is primarily absorbed in the enterocytes and then packaged into chylomicrons to transport into the bloodstream and the lymphatic circulation [63]. Some of the chylomicrons are recognised by adipose tissues, where the chylomicrons are hydrolysed into fatty acids and chylomicron remnants by lipoprotein lipase (LPL) [67]. However, endogenous lipids are mainly synthesised from de novo lipid synthesis in the liver. These lipids are formed into very low‐density lipoproteins (VLDLs). Subsequently, VLDLs can be captured by adipose tissues and metabolised into fatty acids and VLDL remnants (called IDLs) by LPL. Finally, IDLs can bind to the IDL receptor in the liver and then be transformed into low‐density lipoproteins (LDLs) [68]. Moreover, by assembling with apolipoprotein A‐I, free cholesterol is formed into high‐density lipoproteins (HDLs) [69]. HDLs play an essential role in anti‐atherogenic activities, particularly in reverse cholesterol transport, which is defined as the process by which excess cholesterol is turned back from the peripheral tissues, conveyed to the liver and metabolised into bile and faeces [70].

#### 3.1.2 Macrophages and Cholesterol Efflux

**FIGURE 2:**
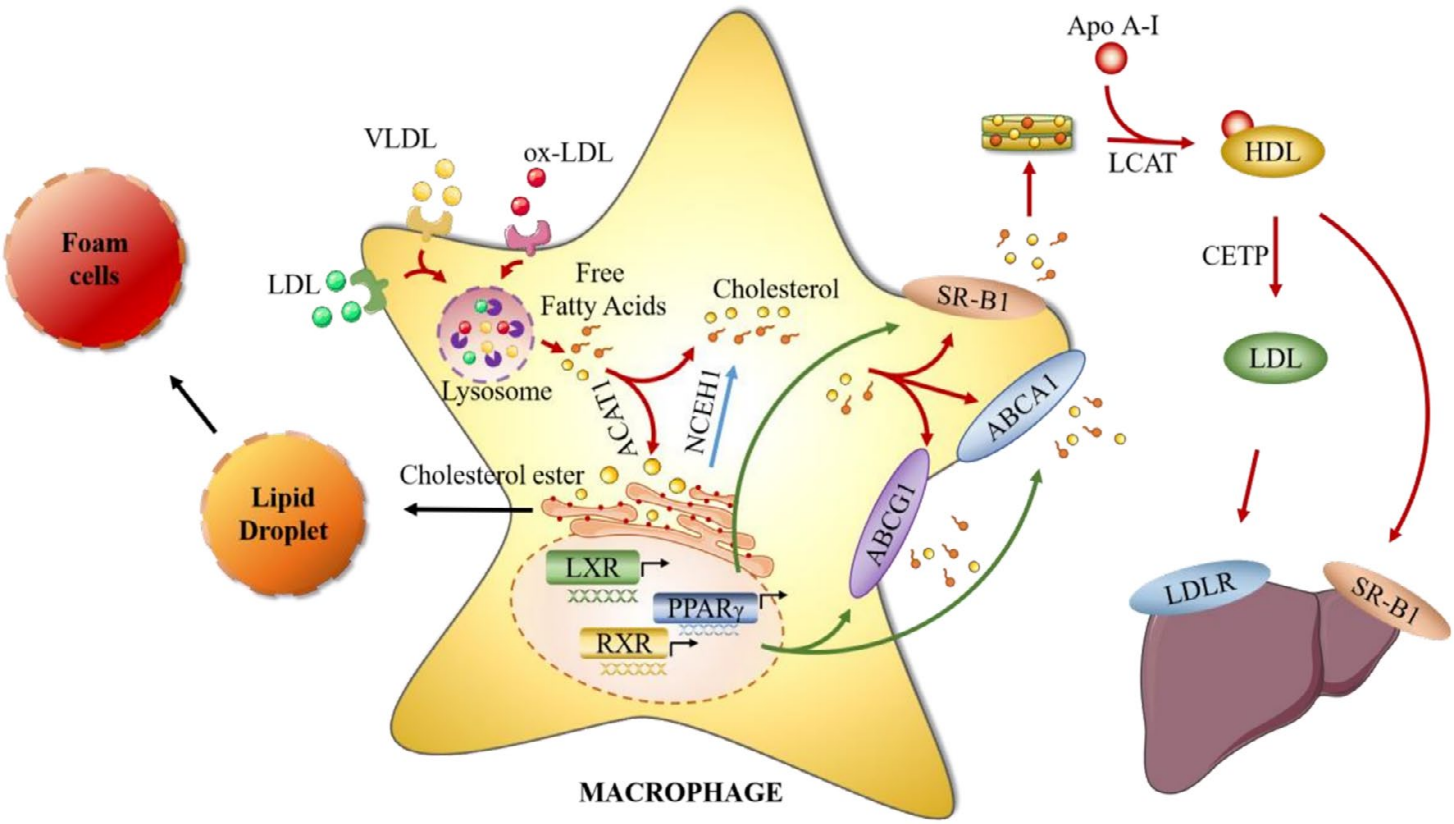
The mechanism of macrophage‐regulated cholesterol efflux.

High total or LDL cholesterol, low HDL cholesterol, and high triglycerides are known as dyslipidemia, which is one of the risk factors for MS and atherosclerotic cardiovascular disease [71, 72]. Macrophages are known to process peripheral cholesterol by regulating cholesterol efflux, which leads to the important role of macrophages in lipid metabolism (Figure 2) [73]. Macrophages have the ability to capture and engulf lipid metabolites, such as LDL, VLDL, ox‐LDL and HDL, via the scavenger receptor of the macrophages [74, 75, 76, 77]. Subsequently, the engulfed lipid will be degraded into fatty acids and cholesterol in the lysosome by lysosomal acid lipase [78]. In general, some of the free cholesterol will be converted to cholesteryl ester by acetyl‐CoA acyltransferase‐1 (ACAT1) in the endoplasmic reticulum, preventing the accumulation of superfluous free cholesterol in the macrophages [79], and then they will either exist in the form of cholesteryl ester stored in lipid droplets and finally promote the formation of foam cells, or flow into the cytosol via neutral cholesterol ester hydrolase 1 (NCEH1) and be subsequently liberated out of macrophages via cholesterol transports [74, 80, 81]. An in vivo experimental study has suggested that the Ldl^−/−^ mice, losing both ACAT1 and NCEH1 in the bone marrow‐derived macrophages, displayed a lower level of cholesterol esters than those losing NCEH1, revealing the essential function of ACAT1 and NCEH1 in the synthesis and hydrolysis of macrophage‐regulated cholesterol esters [82]. In addition, blocking out macrophage‐specific ACAT1 in the high‐fat diet‐induced mice leads to decreased pro‐inflammatory macrophage infiltration in adipose tissues, promotes M2 phenotype transition from M1, and suppresses high‐fat diet‐induced obesity [83]. Studies have also shown that knocking out ACAT1 decreases inflammatory macrophage content and inhibits the development of atherosclerosis without side effects [84]. These studies suggest that decreased cholesterol efflux induces the deposition of cholesterol esters in macrophages and further promotes foam cell formation, revealing the relationship between macrophage‐regulated cholesterol efflux and the development of atherosclerotic cardiovascular disease and MS.

In macrophages, free cholesterol is also conveyed to cholesterol transporters, such as ATP‐binding cassette transport A1 (ABCA1), G1 (ABCG1) and scavenger receptor B class type 1 (SR‐B1), which then flow out of the macrophages and assemble with extracellular lipid‐free apolipoproteins A‐I and HDL [85, 86, 87]. ABCA1^−/−^ ABCG1^−/−^ mice fed with a high‐cholesterol diet and isolated macrophages have shown that knocking out ABCA1 and ABCG1 of macrophages displayed decreased levels of HDL, apolipoproteins A‐I, and apolipoproteins E, and further increased release of inflammatory cytokines and chemokines, which leads to dysfunction of macrophage‐mediated cholesterol efflux, promotion of foam cell formation, and atherosclerosis acceleration [88]. Recently, Zulong found visceral adipose tissue‐derived exosomes in high‐fat diet‐fed obese mice promoted macrophage foam cell formation and M1 macrophage transition, decreased macrophage‐regulated cholesterol efflux via downregulation of ABCA1 and ABCG1, and further exacerbated atherosclerosis [89]. Furthermore, several transcription factors have been proven to activate ABCA1, ABCG1 and SR‐B1 to promote the activity of cholesterol efflux, including nuclear receptors like the liver X receptor (LXR), retinoid X receptor (RXR) and PPARγ [90]. In the cytosol, the released free cholesterol will be esterified by the lecithin cholesterol acyltransferase (LCAT) and eventually mature into HDL [91]. When LCAT is deficient, it leads to the accumulation of cholesterol, which not only increases cell toxicity but also stimulates Kupffer cells to induce hepatic inflammation and the development of metabolic diseases [92, 93, 94]. The level of HDL in the blood is correlated with the development of cardiovascular disease and MS [95]. Due to the exchange of cholesterol esters, triglyceride, and phospholipids among HDLs and LDLs via plasma factors such as the cholesterol ester transfer protein (CETP), finally, triglyceride‐enrich HDLs and cholesterol esters‐enrich LDLs will be transported to the liver via SR‐B1 or LDL receptors [91, 96].

#### 3.1.3 Macrophages and Fatty Acids

In general, fatty acids consumed from high‐fat foods are stored primarily in adipose tissue, not in the liver and skeletal muscle. However, chronic intake of a high‐fat diet can lead to an excess flow of fatty acids into adipose tissue. When the storage capacity of adipose tissue is saturated, the excess fatty acids stay in the bloodstream and thus accumulate in non‐adipose tissue (e.g., liver and skeletal muscle), which is called ectopic fatty acid accumulation [97]. These free fatty acids can bind to macrophages via TLR4 and further initiate the NF‐κB/NLRP3 signalling pathway, leading to the massive secretion of IL‐1β [98]. Moreover, free fatty acids induce dysfunction of mitochondrial fuel switching in macrophages due to acute substrate competition and insatiability of synthase activity, resulting in elevated membrane potential, ROS generation, and lysosome damage [99, 100]. In the liver, Kupffer cells can be activated by adipose tissue‐released fatty acids via the sensor of Kupffer cells, such as TLR1/2/4/6, to generate inflammatory cytokines (e.g., IL‐6, IL‐1β, IL‐10 and TNF‐α) [101, 102]. TNF‐α promotes the de novo lipogenesis and accumulation of fatty acids in hepatocytes by binding to the TNFR1 receptor [101]. In addition, Kupffer cells produce large amounts of IL‐1β, which suppresses PPARα activity to accelerate the accumulation of fatty acids and the development of hepatic steatosis [103].

However, different types of fatty acids have different effects on macrophage polarisation. For instance, a high‐fat diet‐fed animal study has identified that saturated fatty acids primarily switch Kupffer cells into the M1 macrophage phenotype, leading to high secretion of iNOS2, IL‐6, and TNF‐α [104]. On the contrary, when treated with unsaturated fatty acids, Kupffer cells are induced into an M2‐predominant phenotype via activation of PPARγ and NF‐κB signalling pathways. Additionally, the study also proposed that PPARγ serves as a significant regulator for unsaturated fatty acid‐induced macrophage M2‐type polarisation [104]. Omega‐3 fatty acids are unsaturated fatty acids with anti‐inflammatory properties that suppress NLRP3 inflammasome‐induced IL‐1β secretion in macrophages [105]. Docosahexaenoic acid, a polyunsaturated fatty acid, has been shown to activate M2 macrophage polarisation via the p38 MAPK signalling pathway and autophagy [106].

#### 3.1.4 Macrophages and Adipokines

Recently, research has indicated that adipose tissues serve as an endocrine organ to regulate insulin sensitivity, glucose homeostasis, and lipid metabolism by secreting multiple adipokines such as leptin, resistin, adiponectin, and so on [107]. Surging evidence has suggested that these adipokines influence macrophage‐regulated lipid metabolism and inflammation, which are involved in the pathogenesis of MS [108].

In white adipose tissues, insulin can promote the secretion of leptin, which can improve insulin sensitivity, suppress appetite and adipogenesis, and increase lipolysis and fatty acid oxidation [109]. Clinically, the blood concentration of leptin in obese individuals is higher than in lean individuals [110]. Moreover, a high‐fat diet‐fed animal model presents increasing lipid droplet formation in peritoneal macrophages and further enhancement by leptin treatment [111]. Leptin resistance is characterised by increased appetite and less consumption of energy, leading to the development of obesity and cardiovascular disease [112, 113]. Lauar has reviewed the effects of leptin in adipose tissue macrophage infiltration and pro‐inflammatory cytokine secretion in obesity‐induced inflammation, and the mechanism involved in activating the JAK2/STAT3 and PI3K/AKT/mTOR signalling pathways, which further enhance glycolytic enzyme activity, induce mitochondrial dysfunction and promote pro‐inflammatory cytokine release [114]. Subsequently, Lauar has found that leptin can heighten the effects on M1 macrophages, which result in the increasing production of cytokines, promoting glycolysis and changing morphology and function in mitochondria via mTOR signalling pathway activation in LPS‐induced bone marrow‐derived macrophages [115]. Furthermore, the study has also indicated that an increasing level of leptin in macrophages contributes to the development of insulin resistance and an inflammatory response in high‐fat diet‐fed mice [115]. Furthermore, leptin induces macrophage recruitment in the adipocytes [116] and increases the expression of macrophage LPL, a regulated lipid metabolism factor in the development of type 2 diabetes and atherosclerotic cardiovascular disease, through enhancing oxidative stress and activating the PKC signalling pathway [117]. Leptin has also been implicated in macrophage‐mediated cholesterol efflux by activating JAK2 and PI3K‐mediated ACAT1 activation to promote foam cell formation [118].

Resistin, an adipose tissue‐derived hormone in rodents but a pro‐inflammatory cytokine secreted from human macrophages, increases lipid accumulation in oxLDL‐induced macrophages to promote macrophage‐derived foam cell formation [119]. Resistin is involved in the pathogenesis of type 2 diabetes and cardiovascular disease by inducing cholesterol and triglyceride deposition in human macrophages under high glucose conditions [120]. Currently, Bi has demonstrated that resistin can increase lipid accumulation in RAW264.7 macrophages by activating the PPAR‐γ/PI3K/AKT signalling pathway [121].

Adiponectin, an anti‐inflammatory hormone secreted from adipocytes, is negatively correlated with obesity and has a beneficial effect on alleviating atherosclerotic cardiovascular disease [122, 123]. Adiponectin was reported for its ability to facilitate macrophage‐modulated cholesterol efflux by enhancing the affinity of apolipoprotein A‐I to cholesterol to effectively form mature HDL particles [124]. Studies also demonstrated adiponectin can upregulate ABCA1 and ABGC1 protein expression via the PPAR‐γ/LXRα signalling pathway, resulting in increased HDL biogenesis and decreased lipid deposition in macrophage foam cells [124, 125, 126]. Furthermore, in macrophage foam cells, adiponectin can suppress the expression of MCP‐1 and TNF‐α and upregulate SR‐B1 gene expression to enhance lipolysis, thus inhibiting lipid accumulation in macrophages [127]. Currently, C1q tumour necrosis factor‐regulated protein 1 (C1QTNF1), a conserved paralog of adiponectin, can accelerate lipid accumulation in macrophage foam cells by decreasing miR‐454‐5p expression and further activating the Forkhead box O1 (FoxO1)/ABCA1 signalling pathway [128]. Moreover, it was confirmed that adiponectin can promote M2 macrophage polarisation and suppress M1 macrophage activation [129]. Adiponectin can suppress the expression of adiponectin receptors and induce the generation of IL‐12, IL‐6, and TNF‐α in M1 macrophages, but not in M2 macrophages [130].

### 3.2 Insulin Resistance

Insulin, secreted by the pancreatic β cells in response to high blood glucose, plays an important role in energy metabolism, such as inhibiting lipolysis and promoting glucose uptake [131, 132, 133]. Insulin resistance is defined as reduced insulin sensitivity of target organs or tissues, such as the liver, skeletal muscle, and adipose tissue, and is mainly characterised by impaired insulin‐controlled hepatic glucose output in the liver, suppressed insulin‐stimulated glucose intake in skeletal muscle and dysfunctional insulin‐mediated lipogenesis and lipolysis in adipose tissue [134, 135]. Insulin resistance is not only recognised as a key pathogenetic mechanism in MS but has also been shown to be an important contributor to the development of obesity, type 2 diabetes, and cardiovascular disease [136, 137].

Studies have shown that over‐calorie intake‐induced meta‐inflammation is a key trigger for insulin resistance and MS, in which macrophage polarisation plays an important role [138, 139]. Recent studies have proposed that the number of M1 macrophages in the adipose tissue of obese individuals is much higher than that in the adipose tissue of lean individuals and is accompanied by increased levels of IL‐1β, IL‐6, and TNF‐α, which are potent triggers of insulin resistance [15]. Furthermore, in the high‐fat diet‐induced animal models, the number of pro‐inflammatory M1‐like CD11c^+^ hepatic macrophages was significantly higher after transplantation of visceral adipose tissue from obese mice than that from lean mice [140]. In a word, a high‐fat diet promotes macrophage infiltration and M1‐type polarisation in certain organs (hepatic and adipose tissue) and releases large amounts of pro‐inflammatory cytokines, which impair the insulin pathway and ultimately induce the dysfunction of glucose and lipid metabolism.

#### 3.2.1 The Role of Macrophages in Adipose Tissue Insulin Resistance

**FIGURE 3:**
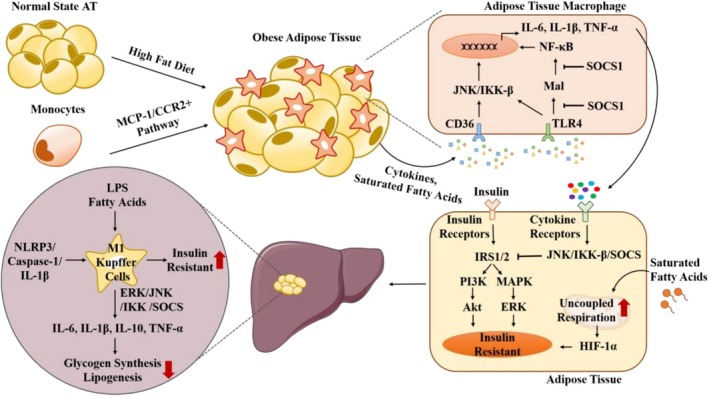
The role of macrophages in adipose tissue insulin resistance and hepatic insulin resistance.

Macrophages are the main immune population in adipose tissue and play an essential role in regulating several physiological processes and maintaining tissue homeostasis, such as phagocytosis of adipocyte debris, mitochondrial uptake from white adipose tissue to macrophages, and insulin sensitivity [141, 142]. Emerging studies in high‐fat diet animal models have confirmed that obesity‐induced low‐grade systemic inflammation is initiated by the activation of Toll‐like receptor 4 (TLR4) in macrophages [143, 144]. Moreover, compensatory angiogenesis often mismatches the hypertrophic expansion of adipose tissue in obese individuals, leading to adipose tissue hypoxia and adipocyte death, followed by the release of pro‐inflammatory cytokines and saturated fatty acids by adipocytes that interact with the scavenger receptors CD36 or TLR4 in adipose tissue macrophages to promote macrophage recruitment and M1 phenotype activation [8, 145] (Figure 3). Meanwhile, activated macrophages secrete monocyte chemoattractant protein 1 (MCP‐1), which promotes a large number of monocytes to be recruited into the obesity‐associated adipose tissue in humans and mice by binding with chemokine (C‐C motif) receptor 2 on the surface of monocytes, further differentiated into macrophages and polarised into pro‐inflammatory M1 macrophages [146, 147]. In activated macrophages, c‐jun N‐terminal kinase (JNK) and IκB kinase β (IKK‐β) signalling pathways are activated to induce inflammatory gene expression, such as IL‐6, IL‐1β and TNF‐α [8, 148, 149, 150]. These pro‐inflammatory cytokines released by M1 macrophages recognise cytokine receptors in adipocytes and subsequently activate the JNK and IKK‐β signalling pathways, leading to inhibition of the downstream insulin receptor substrates (IRS) and the PI3K/AKT or MAPK/ERK pathway, which can impair the insulin sensitivity of adipose tissue [8, 149, 150, 151].

Furthermore, suppressors of cytokine signalling (e.g., SOCS1, SOCS2 and SOCS3) are also associated with macrophage‐mediated adipose tissue insulin resistance. For example, SOCS1 can directly blunt the LPS‐induced inflammatory response by inhibiting the MyD88‐adaptor‐like (Mal) and NF‐κB signalling pathways in adipose tissue macrophages and subsequently decreasing the released inflammatory cytokines, leading to increased insulin sensitivity [152]. In addition, SOCS1 can also enhance the PI3K/AKT signalling pathway, which promotes arginase I expression in IL‐4‐induced M2 macrophages [153]. However, high‐fat diet‐fed SOCS2^−/−^ mice exhibited higher insulin sensitivity and lower cytokine secretion, but adiposity increased, with both M1 macrophages and M2 macrophages augmenting, suggesting that SOCS2 plays a different modulator in the regulation of obesity‐induced chronic inflammation [154]. Notably, in M1 macrophages, SOCS3 is highly expressed, which can inhibit the PI3K signalling pathway and promote the expression of iNOS to inhibit adipose tissue insulin sensitivity [155]. Furthermore, microRNAs have been identified as significant regulators of macrophage M1/M2 polarisation in MS and metabolic disorder‐associated cancers, including colorectal and liver cancer [156, 157, 158, 159, 160, 161, 162]. For example, in vitro and in vivo studies demonstrated that exosomal miR‐29a derived from adipose tissue macrophages decreased insulin sensitivity by reducing PPAR‐δ expression [163, 164].

As mentioned above, in obesity patients or high‐fat diet‐fed obesity animal models, adipose tissue expansion can cause hypoxia. Notably, emerging evidence suggests that HIF proteins are important for macrophage‐mediated adipose tissue insulin resistance [165, 166, 167]. In mouse adipose tissue, saturated fatty acids can trigger HIF‐1α expression via increasing adipocyte oxygen consumption through binding to ADP/ATP translocase 2 (ANT2) to enhance uncoupled respiration in mitochondria, which will result in adipose fibrosis, inflammation and insulin resistance [145, 168]. In adipose tissue macrophages, macrophage‐specific HIF‐1α knockout in mice has not found that HIF‐1α plays an essential role in the activation of adipose tissue macrophages at the early stages of obesity [169]. However, knocking out prolyl‐hydroxylase 2 (PHD2), the enzyme that hydroxylates HIF‐1α in high‐fat diet‐induced mice displays adipose tissue inflammation and insulin resistance, while knocking out interleukin‐1 receptor‐associated kinase‐M (IRAKM) in the mice, a downstream target of HIF‐1α, can reduce macrophage infiltration and M1‐like macrophage numbers [170]. Recently, studies have shown that adipocyte‐derived lactate promotes the activation of inflammatory macrophages by binding with PHD2 and leading to the stabilisation of HIF‐1α, revealing a positive relationship between lactate‐induced insulin resistance and adipose macrophages [171]. Furthermore, macrophage HIF‐2α can inhibit FAO by binding to the CPT1A promoter, resulting in the suppression of the NLRP3 inflammasome and then alleviation of insulin resistance in adipose tissue [172].

#### 3.2.2 The Role of Macrophages in Hepatic Insulin Resistance

In the liver, macrophages are the most proportional immune cells that maintain hepatic functional homeostasis and consist of Kupffer cells and monocyte‐derived macrophages [173]. Similar to adipose tissue macrophages, Kupffer cells play an important role in the development of hepatic insulin resistance. Animal studies have provided evidence that Kupffer cell depletion can enhance hepatic insulin sensitivity under a high‐fat diet and ameliorate inflammation by decreasing the levels of TNF, TLR2, TLR4 and CD14 [174, 175]. In addition, the gut‐liver axis plays a significant role in the activation of Kupffer cell‐mediated insulin resistance. Studies have proposed that a high‐fat diet impairs the epithelial barrier and enhances intestinal permeability and systemic inflammation via remodelling the gut microbiome in obese animals and obese patients [176, 177]. Subsequently, a surge in LPS levels, caused by the disturbance of the microbial gut and enhanced intestinal permeability, can activate pattern recognition receptors of the hepatic macrophages, such as Toll‐like receptors and nucleotide‐binding oligomerization domain‐like receptors [173]. Recognising the foreign substance, Kupffer cells are polarised into the M1 phenotype and eventually secrete cytokines such as IL‐6, IL‐1β and TNF‐α that inhibit insulin signalling by activating the ERK, JNK, IKK and SOCS signalling pathways in hepatocytes, leading to inhibition of glycogen synthesis, lipogenesis, and the promotion of gluconeogenesis [102]. For example, Kupffer cell‐derived inflammatory cytokines, such as TNF‐α and IL‐1β, can induce the serine phosphorylation of IRS‐1 by activating the JNK or ERK signalling pathway and further inhibit the PI3K signalling pathway, leading to insulin resistance [178, 179, 180]. Kupffer cell‐specific NF‐κB knockout mice displayed decreased inflammatory cytokine secretion and enhanced insulin sensitivity [181]. Additionally, primary hepatocytes co‐cultured with Kupffer cells displayed lower insulin sensitivity under the treatment of high glucose [182]. The associated mechanism is involved in the up‐regulation of NLRP3/Caspase‐1/IL‐1β signalling pathways in activated Kupffer cells, which further triggers the NF‐κB signalling pathway and decreases insulin sensitivity in hepatocytes [182].

### 3.3 Oxidative Stress

Oxidative stress is characterised by an imbalance between ROS and the anti‐oxidative system and has been viewed as one of the potential etiologies of MS, particularly in hyperlipidemia and hypertension [183, 184]. Extracellular ROS‐induced inflammation serves as an essential mediator for macrophage recruitment, activation, and foam cell formation, which are key contributors to cardiovascular disease [185]. In the development of cardiovascular disease, macrophages are effector cells, and ROS plays four key functions: (1) Several oxidative enzyme systems such as NADPH oxidase, xanthine oxidase, cyclooxygenases, lipoxygenases, and cytochrome P450 lead to the inactivation of NO, leading to impaired endothelial cells through increasing superoxide anion [186]. (2) ROS can activate the NF‐κB signalling pathway to increase the expression of adhesion molecules and chemokines, thereby damaging endothelial cells [186]. For example, monocyte chemoattractant protein‐1, a kind of adhesion molecule, participates in the inflammatory damage of endothelial cells and facilitates the migration of monocytes to the sub‐endothelium, where they mature into macrophage foam cells [187]. (3) ROS can oxidise vascular LDLs into ox‐LDLs, which is an important contributor to the pathogenesis of atherosclerosis [185, 188]. Monocyte‐derived macrophages can engulf ox‐LDL by binding to its scavenger receptor, resulting in foam cell formation and plaque development, which contribute to atherosclerotic lesions [185, 189]. (4) ROS is a trigger for scavenger receptor expression in smooth muscle cells and also induces their transformation into foam cells [190]. Indeed, the formation of foam cells is attributed to macrophage‐regulated cholesterol efflux dysfunction and is the initial marker of the development of atherosclerosis. A novel study has shown that knocking out TP53‐induced glycolysis and apoptosis regulator (TIGAR), a p53‐inducible gene that can inhibit glycolysis and decrease oxidative stress by enhancing NADPH and ribose production, inactivates the ROS/CYP27A1/LXRα signalling pathway and further augments the lipid deposition in macrophage foam cells [191].

In general, extracellular ROS plays an important role in mediating the phagocytosis of dying cells by macrophages and pro‐inflammatory macrophage polarisation [192, 193]. In the microenvironment, M1 and M2 macrophages play different effects on atherosclerotic plaque: M1 macrophages dominantly distribute in vulnerable plaque regions and contribute to the development of atherosclerosis, while M2 macrophages predominantly appear in stable plaque regions and play a protective effect on atherosclerosis [194, 195, 196]. Except for M1 and M2 macrophages, it is established that other macrophage phenotypes are detected in plaque, such as M4 (in response to CXCL4), Mox (in response to oxidised phospholipids) and Mhem (in response to heme) [197]. Moreover, Mox macrophages perform antioxidant effects via overexpression of Nrf2‐ossiaciated genes such as heme oxygenase‐1 and sulforedoxin‐1 [197, 198]. Furthermore, current studies show that mitochondrial mitofusin 2 (Mfn2) plays an essential role in ROS generation and pro‐inflammatory cytokine release in LPS‐induced macrophages [199]. Deficiency of MFN2 causes mitochondrial membrane potential impairment and a decrease in ROS production, further damaging the generation of TNF‐α, IL‐6, IL‐1β and NO [199]. Interestingly, the study shows that the absence of MFN2 also induces macrophage phagocytosis dysfunction, a switch from M1 to M2 macrophage phenotype. Thereby, improving ROS clearance and promoting macrophages to polarise into the M2 phenotype can be desirable for MS treatment.

Hypertension is another obvious feature of MS [184], and the macrophage‐generated ROS mechanism is thought to play an essential role in the development of hypertension [200]. A high‐fat diet is known to accelerate the development of hypertension in animals, which is involved in increasing mitochondrial ROS levels, aggravating oxidative stress and enhancing angiotensin II receptor type 1 and angiotensin II levels in the kidneys and bloodstream [201]. In the pathogenesis of hypertension, the renin‐angiotensin‐aldosterone system is activated and secretes angiotensin II, binding with the angiotensin II receptor type 1 on the surface of macrophages, which leads to M1 macrophage polarisation and increases M1 macrophage‐derived ROS and inflammatory cytokine levels [200]. These ROS and cytokines will cause vascular endothelial impairment and further block the excretion of renal sodium, resulting in hypertension [202].

## 4 Phytochemicals That Target Macrophages for MS Treatment

**FIGURE 4:**
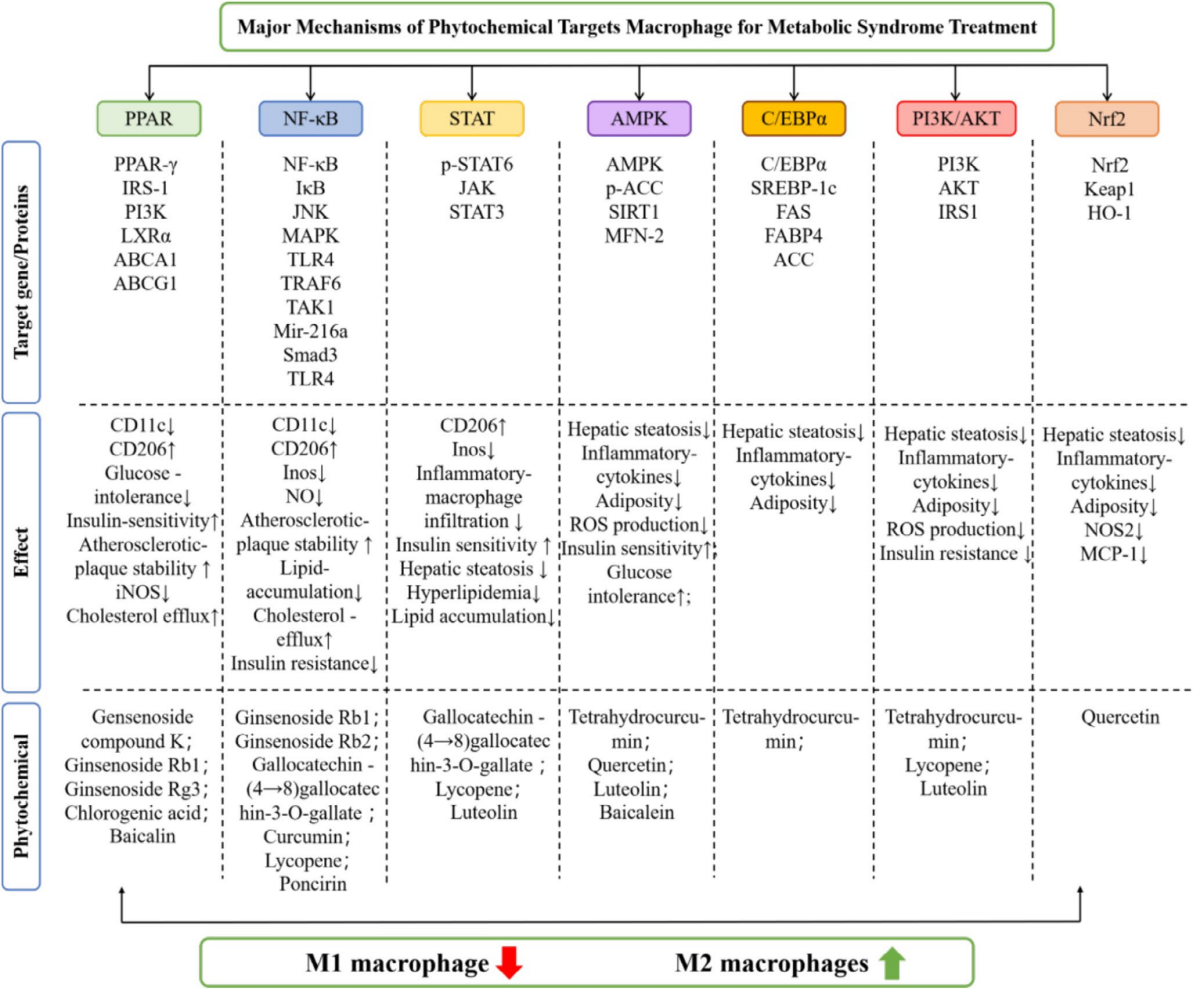
Major mechanism of phytochemical targets macrophages for MS treatment.

Recently, phytochemicals have attracted the attention of scientists for their protective properties, effective therapeutic properties, and safety, and have become popular drug candidates for the treatment of MS [203, 204]. Compared to existing treatments for MS, phytochemicals possess distinctive features and advantages in three aspects: (1) Phytochemicals are natural compounds derived from plants, vegetables, fruits and medicinal herbs, which typically have fewer toxic side effects. This characteristic helps to mitigate the adverse effects and drug–drug interactions often associated with chemotherapy. (2) Natural products containing anti‐inflammatory, anti‐diabetic, anti‐hypertensive or anti‐hyperlipidemic properties are increasingly recognised as effective alternative therapies for the prevention and treatment of MS, type 2 diabetes and cardiovascular disease. (3) Phytochemicals are regarded as valuable options for adjuvant therapy or personalised treatment for patients with MS [205]. Growing evidence proves that various phytochemicals play a role in the treatment of MS, and that macrophage‐regulated metabolic inflammation is one of its key mechanisms. Based on these considerations, we summarise the emerging evidence for phytochemicals targeting macrophages for the treatment of MS (Table 1) (Figure 4).

**TABLE 1:** Phytochemical targets for macrophage MS treatment.

S.No.	Plant origin	Phytochemical	Model in vitro/in vivo	Effect	Macrophage phenotypes	Molecular mechanism	Reference
1	Ginseng	Ginsenoside compound K	Co‐cultured cell model (RAW264.7 and palmitic acid‐induced 3 T3‐L1)	TNF‐α↓; MCP‐1↓; IL‐10↑; CD11c↓; CD206↑	M1 macrophages↓; M2 macrophages↑	PPAR‐γ/IRS‐1 signalling pathway↑; NF‐κB/IκB signalling pathway↓;	[208]
			High‐fat diet‐induced obese mice	M1 inflammatory factors in serum and adipose tissue↓; Insulin sensitivity↑; CD11c↓; CD206↑		PPAR‐γ↑; TLR4/TRAF6/TAK1/NF‐κB signalling pathway↓; IRS1/PI3K/AKT signalling pathway↑	[209]
2	Ginseng	Ginsenoside Rb1	Co‐cultured cell model (RAW264.7 and adipose‐derived stem cells)	MCP‐1↓; TNF‐α↓; IL‐1β ↓; IL‐10↑; CD11c↓; CD206↑	M1 macrophages↓; M2 macrophages↑	PPAR‐γ/IRS‐1/PI3K signalling pathway↑; NF‐κB/IκB signalling pathway↓	[212]
			LPS‐induced Peritoneal macrophages	Arg‐1↑; CD206↑; iNOS↓; MMP‐9↓; IL‐4 ↑; IL‐13↑; IL‐10↑		p‐STAT6 ↑	[213]
			Apoe^−/−^ mice	Lipid accumulation↓; Macrophages infiltration↓; Atherosclerotic plaque stability↑; MMP‐9↓; Collagen↑; Smcs↑; Arg‐1↑; iNOS↓; IL‐4↑; IL‐13↑			[213]
3	Ginseng	Ginsenoside Rb2	LPS‐induced RAW264.7 cells	TNF‐α↓; IL‐6↓; IL‐1β↓; iNOS↓; NO↓; COX‐2↓	M1 macrophages↓; M2 macrophages↑	IKKβ, NF‐κB, JNK and p38↓; GPR120↑	[216]
			Apoe^−/−^ mice infected with mir‐216a	Atherosclerotic plaque stability↑; Lipid accumulation↓; M1 macrophages infiltration in plaques↓; CD68↓; CD16/32↓; MMP‐9↓; Collagen↑	M1 macrophages↓		[217]
			Ox‐LDL‐ and mir‐216a‐induced THP‐1; Ox‐LDL‐induced PBMC	Lipid accumulation↓; Cell apoptosis↓; TNF‐α↓; MCP‐1↓; CD68↓; CD36↑; SR‐A1↑; Cholesterol efflux↑; ABCG1↑	M1 macrophages↓	Mir‐216a/Smad3/IκBα signalling pathway↓	
4	Ginseng	Ginsenoside Rg3	Advanced glycation end products‐induced macrophages	TNF‐α↓; IL‐6↓; IL‐10↑; TGF‐β↑; CD86↓; CD206↑	M1 macrophages↓; M2 macrophages↑	PPAR‐γ↑	[218]
			Apoe^−/−^ mice	Atherosclerotic plaque stability↑; Lipid accumulation↓; M1 macrophages infiltration in plaques↓; Collagen↑; Smcs↑; CD86↓; CD206↑; Arg‐1↑; iNOS↓			
5	Camellia ptilophylla (Cocoa tea)	Gallocatechin‐(4 → 8)‐gallocatechin‐3‐O‐gallate (proanthocyanidin dimer)	High‐fat diet‐induced obese mice	Epididymal white adipose tissue hypertrophy↓; Inflammatory macrophage infiltration↓; Insulin sensitivity↑; Hepatic steatosis↓; Hyperlipidemia↓; Lipid accumulation↓;6 TNF‐α↓; IL‐6↓; MCP‐1↓; CD11c↓; Leptin↓; Adiponectin↑	M1 macrophages↓	JAK/STAT3 signalling pathway↓; NF‐κB signalling pathway↓	[225]
			TNF‐α‐induced 3 T3‐L1 adipocytes	Lipid accumulation↓; IL‐6↓; MCP‐1↓; COX‐2↓		IKKβ, NF‐κB, JNK and p38↓; PPARγ, C/EBPα, SREBP‐1c, FAS, FABP4, and ACC↓	
6	Green tea	Epigallocatechin‐3‐gallate	High‐fat diet+30% Fructose‐induced obese mice	Body weight↓; Liver weight↓; TC↓; TG↓; Liver enzymes↑	Ly6C^+^ and MHCII^+^ hepatic macrophages↓ CD206^+^, CD23^+^ hepatic macrophages↑;		[225]
			TNF‐α‐induced macrophages	ABCA↑; Cholesterol efflux↑		NF‐κB pathway↓; Nrf2/Keap1 pathway ↑	[226]
7	Green tea	Catechin‐rich green tea extract	High‐fat diet‐induced obese mice	Dyslipidemia↓; Insulin resistance↓; TNF‐α↓; IL‐6↓; MCP‐1↓; iNOS↓; Endotoxemia↓; Gut barrier integrity↑; Obesity‐associated gut dysbiosis↓	M1 macrophages↓	TLR4/NFκB pathway↓	[227]
8	Flos Lonicerae/coffee	Chlorogenic acid	High‐fat diet‐induced obese mice	Diet‐induced weight gain↓; Hepatic steatosis↓; Obesity‐regulated inflammation↓; Glucose tolerance↑; Insulin sensitivity↑; TNF‐α↓; MCP‐1↓; Ccr2↓; Mgat1↓; CD36↓; Fabp4↓	F4/80 cells in epididymal white adipose tissue↓; CD68 cells in epididymal white adipose tissue↓; CD11b cells in epididymal white adipose tissue↓; CD11c cells in epididymal white adipose tissue↓	Hepatic PPARγ pathway↓	[231]
9	Turmeric	Curcumin	*ob/ob* mice	Insulin resistance↓; TNF‐α↓; IL‐6↓; IL‐4↑	M1 macrophages↓	NF‐κB pathway↓	[234]
			High‐fat high‐sugar diet‐induced mice	Steatohepatitis↓; Liver fibrosis↓; Liver dysfunction↓; Adipocyte size↓; Hyperglycaemia↓; Insulin resistance↓; CCL3↓; CCL4↓; CXCL16↓; CMTM7↓; CMTM3↓; SLIT↓	M1 macrophages↓	JNK‐MAPK‐NF‐κB pathway↓	[235]
10	Turmeric, *Zingiber mioga* , *Zingiber officinale* and *Curcuma zedoaria*	Tetrahydrocurcumin	High‐fat diet‐induced obese mice	Adiposity↓; Inflammatory macrophage Infiltration in mouse epididymal adipose tissues↓; Inflammatory macrophage polarisation in mouse epididymal adipose tissues↓; Hepatic steatosis↓; Inflammatory cytokines↓; F4/80↓; CD11b↓; CD163↑	M1 macrophages↓; M2 macrophages↑	PPARγ, C/EBPα, and FAS↓; Dlk1/Pref‐1↑; AMPK/p‐ACC↑; IRS/PI3K/AKT signalling↑	[237]
11	Tomatoes	Lycopene	LPS‐induced RAW264.7 cells	TNF‐α↓; IL‐6↓; IL‐1β↓	M1 macrophages↓	JNK pathway↓; NF‐κB pathway↓	[242]
			High‐fat diet‐induced obese mice	Glucose intolerance↓; Hyperinsulinemia↓; Adipocyte hypertrophy↓; Macrophage infiltration in Epididymal white adipose tissue↓; Hepatic steatosis↓; Pro‐inflammatory cytokines and chemokines↓; ROS production↓	M1 macrophages↓; M2 macrophages↑	p‐AKT in the adipose tissue and liver↑	[243]
			LPS‐ induced RAW 264.7 macrophages; IL‐4 induced RAW 264.7 macrophages	TNF‐α↓; IL‐1β↓; CCL2↓; CCL5↓; Arg‐1↑; Chi3l3↑; IL‐10↑		NF‐B/MAPK pathway↓; STAT6/AKT pathway↑	[243]
12	Many vegetables and medicinal herbs contain quercetin	Quercetin	High‐fat diet‐induced obese mice	Body weight↓; Insulin sensitivity↑; Glucose intolerance↑; Mast cells and macrophage infiltration↓; Pro‐inflammatory cytokines↓; Leptin↓; Adiponectin↓; Glut4↑	M1 macrophages↓; M2 macrophages↑	AMPKα1/SIRT1 pathway ↑	[247]
			High‐fat diet‐induced obese mice	TNF‐α↓; IL‐6↓; IL‐10↑; Arg‐1↑; Mrc1↑; NOS2↓; MCP‐1↓	M1 macrophages↓; M2 macrophages↑	Nrf2/HO‐1↑	[248]
13	Many vegetables and medicinal herbs contain luteolin	Luteolin	Postmenopausal obese mice	Insulin resistance↓; CD11c^+^ adipose tissue macrophages↓; Macrophage infiltration↓; CD11c↓; MCP‐1↓; TNF‐α↓; IL‐6↓	M2 macrophages↑	CD11c^+^ adipose tissue macrophages in gonadal adipose tissue↓	[252]
			LPS‐induced RAW 264.7 macrophages	CD11c↓	M1 macrophages↓	Antagonise or alleviate oestrogen deficiency‐induced inflammation	
			LPS‐induced RAW 264.7 macrophages	ROS↓; Apoptosis‐associated speck‐like protein containing CARD↓; IL‐18↓; IL‐1β↓; Arg‐1↑; IL‐10↑; TNF‐α↓; IL‐6↓; iNOS↓	M2 macrophages↑; M1 macrophages↓	NLRP3/caspase‐1 pathway↓	[255]
			Ang II‐induced RAW 264.7 macrophages	Apoptotic rate↓; Arg‐1↑; IL‐10↑; CD206↑; Dectin‐1↑; TNF‐α↓; IL‐6↓; iNOS↓; CD16/32↓	M2 macrophages↑; M1 macrophages↓	Bcl‐2/Bax/capase‐3 pathway↓; PI3K/AKT pathway↓	[253]
			High‐fat diet‐induced obese mice	Insulin resistance↓; Inflammatory macrophage infiltration↓; CD36 and Plin2 in epididymal adipose tissues↓	M1 macrophages↓; Metabolic activation macrophages↓	AMPKα1 signalling↑	[251]
			LPS‐/IFN‐γ induced RAW 264.7 macrophages; IL‐4 induced RAW 264.7 macrophages	CD86↓; CD206↑; IL‐6↓; iNOS↓; IL‐1β↓; Arg‐1↑; CD163↑; IL‐10↑	M1 macrophages↓; M2 macrophages↑	p‐STAT3 ↓; p‐STAT6↑	[254]
14	*Scutellaria baicalensis* Georgi	Baicalin	LPS‐induced RAW 264.7 macrophages	IL‐6↓; G‐CSF↓; VEGF↓; Macrophage inflammatory proteins (MIP‐1α，MIP‐1β，MIP‐2, PANTES)↓; ROS↓; Inflammatory genes (Chop, Fas, Nos, Ptgs2, Stat1, c‐Jun, c‐Fos, At1a)↓	M1 macrophages↓	Calcium‐CHOP pathway↑	[262]
			High‐fat diet‐induced rabbit atherosclerosis	Atherosclerotic lesion sizes↓; Lipid accumulation↓		PPARγ/LXRα/ABCA1/ABCG1 pathway↑	[261]
15	*Scutellaria baicalensis* Georgi	Wogonin	Ox‐LDL‐induced murine J774.A1 macrophages	Cholesterol accumulation↓; Cholesterol efflux↑		PP2B‐mediated ABCA1 stability↑	[263]
16	*Scutellaria baicalensis* Georgi	Baicalein	Ox‐LDL‐induced THP‐1 macrophages	Cholesterol efflux↑; TNF‐α↓; IL‐6↓; IL‐1β↓		PPARγ/LXRα/ABCA1/ABCG1 pathway↑	[265]
			Ox‐LDL‐induced/Ang II‐induced RAW 264.7 macrophages	TNF‐α↓; IL‐6↓; PAI‐1↓; MMP‐9↓		MAPK/NF‐κB pathway↓; AMPK/MFN‐2 pathway↑	[264]
17	*Scutellaria baicalensis* Georgi	Root of Scutellaria baicalensis Georgi ethanol extract	High‐fat diet‐induced obese mice	Insulin resistant↓; Fasting and postprandial glucose↓; Fasting insulin↓; HOMA‐IR↓; Triglycerides↓; Low‐density lipoprotein cholesterol↓; Epididymal fat weight↓; Liver weight↓; Adipose tissue macrophages↓	M2 macrophages↑; CD11b + Kupffer cells↓	TNF‐α↓; F4/80 Kupffer cells↓	[266]
18	*Poncirus trifoliata* Rafinesque	Poncirus fructus	High‐fat diet‐induced obese mice	Insulin resistant↓; Fasting and postprandial glucose↓; Fasting insulin↓; HOMA‐IR↓; Total‐cholesterol↓; Triglycerides↓; Low‐density lipoprotein cholesterol↓; Fat accumulation↓; Adipose tissue macrophages↓	M2 macrophages; F4/80 + Kupffer cells↓; CD68 + Kupffer cells↓; CD11b + Kupffer cells↓	TNF‐α↓; F4/80 Kupffer cells↓	[268]
19	*Poncirus trifoliata* Rafinesque	Poncirin	LPS‐induced RAW 264.7 macrophages	iNOS↓; COX‐2↓; TNF‐α↓; IL‐6↓	M1 macrophages↓	NF‐κB activity↓	[269]

### 4.1 Ginsenosides

#### 4.1.1 Ginsenoside Compound K

Ginsenoside compound K is the major deglycosylated metabolite from ginsenoside, one of the rare ginsenosides with anti‐diabetic, anti‐inflammatory, anti‐angiogenesis and anti‐colorectal cancer activities [206, 207]. Recent studies have shown that ginsenoside compound K can effectively decrease the levels of TNF‐α and MCP‐1 and increase IL‐10 in the co‐culture models of RAW264.7 and palmitic acid‐induced 3 T3‐L1 cells [208]. Furthermore, ginsenoside compound K not only suppresses the migration of RAW264.7 but can also promote M2 macrophage polarisation and inhibit M1 macrophage polarisation by activating the PPAR‐γ/IRS‐1 signalling pathway and suppressing the NF‐κB/IκB signalling pathway [208]. In a high‐fat diet‐induced obesity mouse model, ginsenoside compound K can reduce the expression of pro‐inflammatory factors such as MCP1, TNF‐α and IL‐1β in serum and adipose tissue, improve insulin sensitivity, and inhibit M1 macrophage activation by activating the PPAR‐γ/IRS1/PI3K/AKT signalling pathway and inhibiting the TLR4/TRAF6/TAK1/NF‐κB signalling pathway [209]. In a randomised, double‐blind, placebo‐controlled clinical trial, 42 subjects with impaired fasting glucose or type 2 diabetes who were treated with fermented red ginseng (enriched in ginsenoside compound K) demonstrated a decrease in postprandial blood glucose levels and an increase in postprandial insulin levels compared to the placebo group. These findings suggest that fermented red ginseng (enriched in ginsenoside compound K) may have a regulatory effect on hyperglycemia [210].

#### 4.1.2 Ginsenoside Rb1

Ginsenoside Rb1, another major ingredient in *Panax ginseng* C. A. Mey., is reported to possess anti‐diabetic, anti‐hypertensive and anti‐hyperlipidemic properties [211]. Recent evidence has shown that ginsenoside Rb1 can reduce the expression of MCP‐1, TNF‐α and IL‐1β and increase the level of IL‐10 in the co‐culture models of RAW264.7 and adipose‐derived stem cells through activation of the PPAR‐γ/IRS‐1/PI3K signalling pathway and inhibition of the NF‐κB/IκB signalling pathway [212]. These results suggest ginsenoside Rb1 alleviates insulin resistance in the co‐culture model of adipose‐derived stem cells and primary macrophage by promoting M2 macrophage activation and preventing M1 macrophage polarisation. In addition, ginsenoside Rb1 treatment significantly increases the M2 macrophage markers (such as Arg‐1 and CD206) and reduces the M1 macrophage marker iNOS in the LPS‐induced peritoneal macrophages [213]. The study also reports that ginsenoside Rb1 decreases lipid accumulation and improves atherosclerotic plaque stability by promoting M2 macrophage polarisation in ApoE^−/−^ mice [213].

#### 4.1.3 Ginsenoside Rb2

Ginsenoside Rb2 is a protopanaxadiol‐type saponin widely distributed in the stems and leaves of *Panax ginseng* C. A. Mey., with strong antioxidant, anti‐inflammatory, and anti‐colorectal cancer effects [214, 215]. A recent study suggests that ginsenoside Rb2 can significantly enlarge the inhibitory effect of ω‐3 fatty acid on LPS‐induced inflammatory cytokines (such as TNF‐α, IL‐6 and IL‐1β) and NO secretion in macrophages, suggesting that the mechanism is related to macrophage polarisation 216. Mechanistically, ginsenoside Rb2 amplifies the suppression of IKKβ, NF‐κB, JNK, and p38, and time‐dependently elevates GPR120 mRNA expression, a PPARγ target gene in adipocytes [216]. Another study demonstrates that ginsenoside Rb2 prominently reduces the expression of M1 macrophage markers (such as CD86) and decreases miR‐216a‐induced inflammation by impairing the Smad3/IκBα signalling pathway in Ox‐LDL and miR‐216a‐induced THP‐1 or PBMC [217]. Moreover, ginsenoside Rb2 treatment could inhibit lipid accumulation and M1 macrophage infiltration in plaques and promote atherosclerotic plaque stability in miR‐216a‐infected ApoE−/− mice [217].

#### 4.1.4 Ginsenoside Rg3

Consistent with the bioactive effects of other ginsenosides, ginsenoside Rg3 has also been shown to exert a therapeutic effect on MS through anti‐inflammatory and antioxidant activities [203]. In the advanced glycation end products‐induced macrophage model, ginsenoside Rg3 suppresses inflammatory cytokines and chemokine secretion and promotes M2 macrophage activation by inducing the PPAR‐γ signalling pathway [218]. Additionally, a randomised controlled clinical trial investigated the effects of co‐administering Korean Red ginseng and American ginseng (two species of ginseng that contain with high amounts of ginsenoside Rg3) in patients with type 2 diabetes and hypertension. The study demonstrated an improvement in central systolic blood pressure but found no direct effect on endothelial function. These findings suggest the potential utility of ginseng for managing hypertension, thereby expanding its role in diabetes management [219].

### 4.2 Proanthocyanidins

Proanthocyanidins, natural flavonoids widely distributed in plants, flowers, fruits, and nuts, are also known as condensed tannins with various pharmacological effects, including antioxidant, cardioprotective, immunomodulatory, and antidiabetic properties [220]. Recently, gallocatechin‐(4 → 8)‐gallocatechin‐3‐O‐gallate, a proanthocyanidin dimer isolated from *Camellia ptilophylla* Chang, has been reported to exert protective effects in high‐fat diet‐induced obesity mice and TNF‐α‐induced 3 T3‐L1 adipocytes by inhibiting M1 macrophage activation [221]. Interestingly, a randomised, controlled, crossover trial involving healthy participants with mild hypercholesterolemia (23 women and 17 men) had a mean ± SD BMI of 25.3 ± 3.7 kg/m^2^ and an average age of 51 ± 11 years. This study demonstrated that supplementation with proanthocyanidin‐rich apples led to a significant reduction in serum cholesterol, LDL cholesterol, triacylglycerol levels, and intercellular cell adhesion molecule‐1 (ICAM‐1) [222]. Furthermore, a randomised, single‐blind, crossover clinical trial discovered that the consumption of sorghum drinks containing proanthocyanidins could effectively lower postprandial glycaemia in subsequent meals [223].

### 4.3 Catechins

Catechins are highly active constituents of *Camellia* sinensis, including (‐)‐epicatechin, (‐)‐epicatechin‐3‐gallate, (‐)‐epigallocatechin and (‐)‐epigallocatechin‐3‐gallate [224]. A recent study provided proof that the mice fed a high‐fat diet and 30% fructose presented decreased Ly6C^+^ and MHCII^+^ hepatic macrophages (M1 macrophage markers) and increased CD206^+^ and CD23^+^ hepatic macrophages (M2 macrophage markers) after epigallocatechin‐3‐gallate treatment [225]. Furthermore, in TNF‐α‐induced macrophages, epigallocatechin‐3‐gallate treatment can enhance cholesterol efflux by inhibiting the NF‐κB pathway and up‐regulating the Nrf2/Keap1 pathway [226]. Moreover, catechin‐rich green tea extract has been reported to attenuate the expression levels of TNF‐α, iNOS and MCP‐1 in the adipose tissues of high‐fat diet‐induced mice by a mechanism related to the inhibition of M1 macrophage polarisation [227]. A double‐blind, placebo‐controlled randomised trial involving healthy adults and adults with MS indicated that catechin‐rich green tea extract could improve glycemic control in patients with MS, partly by decreasing gut inflammation and small intestinal permeability [228].

### 4.4 Chlorogenic Acid

Chlorogenic acid, an active component of *Lonicera japonica* Thunb, has been shown to have a therapeutic effect on MS [229, 230]. Chlorogenic acid inhibits high‐fat diet‐induced insulin resistance and hepatic steatosis and decreases high‐fat diet‐induced chronic inflammation and M1 macrophage activation in the liver by activating the hepatic PPARγ pathway [231]. A randomised, double‐blind, placebo‐controlled crossover trial involving healthy participants demonstrated that the continuous intake of co‐administered green tea catechins and coffee chlorogenic acids improved postprandial glycemic control and insulin sensitivity, which are beneficial for the prevention of diabetes [232].

### 4.5 Curcumin

Curcumin, a lipophilic polyphenol derived from *Curcuma longa* L., is revealed to possess various bioactive properties against cancer, type 2 diabetes, and inflammation [233]. Suman et al. reported the anti‐insulin resistance and anti‐inflammation effects of curcumin in *ob/ob* mice and found that the mechanism involved was the inhibition of peritoneal M1 macrophage activation and pro‐inflammatory cytokine secretion through down‐regulation of the NF‐κB pathway [234]. Furthermore, Muralidhara et al. identified curcumin liposomes that could serve as a lipophilic NF‐κB inhibitor to target inflammatory liver macrophages, inhibit hepatic inflammation and lipid deposition, and enhance insulin sensitivity [235]. Tetrahydrocurcumin, the major metabolite of curcumin, is well stabilised in plasma [236]. In high‐fat diet‐induced mice, tetrahydrocurcumin is found to significantly decrease the ratio of M1/M2 macrophages in adipose tissues, improving insulin resistance and hepatic steatosis by reducing M1 macrophage polarisation and promoting M2 macrophage activation [237]. Findings from two double‐blind clinical studies involving individuals with MS for over a 12‐week period demonstrated that curcumin consumption (200 mg daily) improved dyslipidemia but had no effect on body composition, hypertension or glycemic control [238]. However, a randomised, double‐blind, placebo‐controlled clinical study on patients with MS for 12 weeks suggested that curcumin (500 mg daily) improved arterial stiffness and weight management [239]. The different dosages (200 mg daily vs. 500 mg daily) may explain the contrasting conclusions regarding the impact of curcumin on weight in two clinical studies.

### 4.6 Lycopene

Lycopene is a lipophilic antioxidant extracted mainly from tomatoes [240]. Recently, Ni et al. found that dietary lycopene intake was inversely associated with the development of MS, type 2 diabetes, and cardiovascular disease through meta‐analysis [241]. In vitro, lycopene can reduce the expression level of LPS‐induced TNF‐α in RAW264.7 by suppressing the JNK pathway and the NF‐κB pathway [242]. Emerging evidence has shown the anti‐inflammatory and anti‐diabetic effects of lycopene on high‐fat diet‐induced mice and LPS‐stimulated macrophages by reducing pro‐inflammatory cytokines and chemokines through promoting M2 macrophage polarisation [243]. The associated mechanism involved the down‐regulation of the NF‐κBB/MAPK pathway and the up‐regulation of the STAT6/AKT pathway [243]. Consistently, Yage et al. found that lycopene could alleviate hyperglycemia and dyslipidemia in high‐fat diet/streptozotocin‐induced diabetic mice and in Min6 cells exposed to high glucose/palmitic acid‐RAW264.7 conditioned medium, which was associated with M1/M2 macrophage polarisation homeostasis through inhibition of the TLR4/MyD88/NF‐κB signalling pathway [244]. Two clinical trials demonstrated that supplementation with lycopene‐enriched tomato‐juice or red orange juice in volunteers with MS or overweight, respectively, reduced insulin resistance and blood lipid levels [245, 246].

### 4.7 Quercetin

Quercetin is an important flavonoid widely distributed in vegetables, fruits and medicinal herbs, possessing various bioactive effects on MS [204]. Quercetin suppresses mast cells and macrophage recruitment into the epididymis adipose tissues of high‐fat diet‐induced mice and up‐regulates the ratio of M2 macrophage polarisation and down‐regulates M1 macrophage polarisation by activating the AMPKα1/SIRT1 signal pathway [247]. In addition, quercetin could enhance AMPKα1 and SIRT1 protein expression to attenuate M1 macrophage activation and inhibit the pro‐inflammatory response in mouse bone marrow‐derived macrophages [247]. Quercetin can significantly decrease the levels of MCP‐1, TNF‐α and NOS2 (M1 macrophage markers) in co‐cultured lipid‐laden hepatocytes and macrophages, while these effects are reversed by HO‐1 inhibitor treatment and Nrf2 deficiency treatment, indicating the mechanism is related to the Nrf2/HO‐1 signalling pathway [248]. In addition, a double‐blind crossover clinical trial demonstrated that quercetin (150 mg/day) reduced waist circumference, postprandial systolic blood pressure and postprandial triacylglycerol levels, while increasing HDL‐C concentrations in males with the apolipoprotein E genotype [249].

### 4.8 Luteolin

Luteolin is a flavonoid compound widely derived from many vegetables and medicinal herbs, such as broccoli, celery, green pepper and perilla leaves [250]. Some animal experiments have revealed the bioactive effects of luteolin against insulin resistance and inflammatory responses induced by high‐fat diets, and these effects are associated with M2 macrophage polarisation [251, 252]. Other in vitro experiments have shown that luteolin inhibits LPS/Ang‐II‐induced M1 macrophage polarisation and promotes M2 macrophage activation [253, 254, 255]. These findings indicate luteolin can be an essential candidate for macrophage polarisation in the treatment of MS. A clinical trial involving patients with MS confirmed that supplementation with chlorogenic acid and luteolin could improve several important biomarkers, including body weight, waist circumference, plasma lipids, insulin resistance, and HbA1c level [256]. Additionally, another randomised, double‐blind, placebo‐controlled clinical study demonstrated that the co‐administration of chlorogenic acid and luteolin has a therapeutic effect on body weight management, glycaemic control, and lipid metabolism in individuals with pre‐obesity [257].

### 4.9 *Scutellaria baicalensis* Georgi

*Scutellaria baicalensis* Georgi is a commonly used traditional Chinese medicine, rich in a variety of biologically active compounds, such as baicalin, wogonin and baicalein [258]. These phytochemicals have been reported to possess multiple pharmacological activities, including anti‐hyperlipidemia, anti‐hyperglycemia and anti‐hypertension [259, 260]. Research on high‐fat diet‐induced rabbit atherosclerosis has shown that administration of baicalin can attenuate atherosclerotic plaque and lipid accumulation by increasing macrophage‐mediated cholesterol efflux and inhibiting foam cell formation [261]. Baicalin is also effective in suppressing the production of NO, hydrogen peroxide, and pro‐inflammatory cytokines in LPS‐induced RAW 264.7 [262]. The associated mechanism may involve the down‐regulation of pro‐inflammatory gene expression, such as Chop, Stat1 and c‐Jun [262]. Moreover, wogonin or baicalein treatment has also been confirmed to decrease Ox‐LDL‐induced macrophage lipid accumulation and inflammation and promote macrophage‐mediated cholesterol efflux [263, 264, 265]. A recent study by Hyun et al. demonstrated that an ethanol extract of the root of *S. baicalensis* Georgi could attenuate insulin resistance in high‐fat diet‐induced mice by suppressing pro‐inflammatory cytokine production, enhancing M2 macrophage activation in adipose tissue, and down‐regulating CD11b^+^ Kupffer cells [266].

### 4.10 *Poncirus trifoliata* Rafinesque

Poncirus fructus, a dried and immature fruit from *P. trifoliata* Rafinesque, is widely used for the therapy of cancer and digestive diseases [267]. A recent study suggested that Poncirus fructus has an anti‐insulin resistance effect in high‐fat diet‐induced obese mice through attenuating M1 adipose tissue macrophage and Kupffer cell activation and promoting macrophage polarisation into M2 macrophages [268]. Poncirin, a flavanone glycoside extracted from Poncirus fructus, suppresses the expression of iNOS, COX‐2, TNF‐α and IL‐6 in LPS‐induced RAW 264.7 macrophages by inhibiting the NF‐κB signalling pathway to promote M2 macrophage polarisation [269].

## 5 Conclusions

MS has a complex and not yet fully understood pathogenesis. Growing studies highlight the essential roles of macrophage‐mediated metaflammation in MS, which are strongly linked to macrophage polarisation. Emerging research on phytochemicals has demonstrated that natural products can influence the regulation of macrophage phenotypes, particularly M2 macrophages, offering a potential therapeutic avenue for MS. This has motivated us to review the role of macrophage‐mediated metaflammation in the development of MS and to summarise potential natural products that exhibit anti‐inflammatory, anti‐hyperglycaemic, and anti‐hyperlipidaemic properties by targeting macrophage polarisation. In this review, we summarise various phytochemicals that target macrophage polarisation for the treatment of metabolic syndrome, including ginsenoside Rb1, ginsenoside Rb2, ginsenoside Rg3, catechins, curcumin, lycopene, quercetin and luteolin. Additionally, several phytochemicals have undergone multiple clinical trials and have shown excellent clinical efficacy, such as chlorogenic acid, curcumin, quercetin and luteolin. We believe that as interest in herbal medicine continues to grow, more effective drugs will be discovered and applied in clinical practice. By reviewing the effects of phytochemicals on macrophages to treat metabolic syndrome, we found that the main signalling pathways involved are PPAR‐γ, NF‐κB, AMPK and the PI3K/AKT pathway, which also provide targets for subsequent drug development. However, the role of macrophages at every stage of the progression of metabolic diseases has not been fully elucidated, and further clinical trials are necessary to confirm the potential of phytochemical therapies for patients with MS. In conclusion, this review reflects on the role of macrophage‐mediated metaflammation in MS and compiles current research on natural products that may help treat MS by targeting macrophage polarisation. We hope this paper will inspire new ideas for medication development for the treatment of MS.

## Author Contributions

**Zeting Ye:** writing – original draft, writing – review and editing, visualization. **Yanlin Li:** writing – review and editing. **Xiaolin Yang:** writing – review and editing. **Chenglin Li:** writing – review and editing. **Rui Yu:** writing – review and editing. **Guangjuan Zheng:** supervision, validation, funding acquisition. **Zuqing Su:** conceptualisation, supervision, validation, funding acquisition.

## Ethics Statement

This article does not contain any studies with human or animal subjects.

## Conflicts of Interest

The authors declare no conflicts of interest.

## Data Availability

The data that support the findings of this study are available from the corresponding author upon reasonable request.
